# Overexpression of ribosome binding protein 1 (RRBP1) in breast cancer

**DOI:** 10.1186/1559-0275-9-7

**Published:** 2012-06-18

**Authors:** Deepthi Telikicherla, Arivusudar Marimuthu, Manoj Kumar Kashyap, Y L Ramachandra, Sujatha Mohan, Juan Carlos Roa, Jagadeesha Maharudraiah, Akhilesh Pandey

**Affiliations:** 1 Institute of Bioinformatics, International Tech Park, Bangalore 560 066, India; 2 Department of Biotechnology, Kuvempu University, Shankaraghatta 577451, India; 3 Research Unit for Immunoinformatics, RIKEN Research Center for Allergy and Immunology, 1-7-22 Suehiro-cho, Tsurumi-ku, Yokohama, Kanagawa 230-0045, Japan; 4 Department of Pathology, Universidad de La Frontera, Temuco, Chile; 5 Department of Pathology and Laboratory Medicine, Icon Hospitals, Bangalore 560027, India; 6 Manipal University, Madhav Nagar, Manipal 576104, India; 7 McKusick-Nathans Institute of Genetic Medicine, Johns Hopkins University School of Medicine, Baltimore, MD 21205, USA; 8 Department of Biological Chemistry, Johns Hopkins University School of Medicine, Baltimore, MD 21205, USA; 9 Department of Pathology, Johns Hopkins University School of Medicine, Baltimore, MD 21205, USA; 10 Department of Oncology, Johns Hopkins University School of Medicine, Baltimore, MD 21205, USA; 11 McKusick-Nathans Institute of Genetic Medicine, 733 N. Broadway, BRB 527, Johns Hopkins University, Baltimore, MD 21205, USA

**Keywords:** Breast neoplasms, ES130, p180, 180 kDa ribosome receptor homolog, Endoplasmic reticulum membrane protein, Immunohistochemistry, Biomarker, Early detection

## Abstract

The molecular events that lead to malignant transformation and subsequent metastasis of breast carcinoma include alterations in the cells at genome, transcriptome and proteome levels. In this study, we used publicly available gene expression databases to identify those candidate genes which are upregulated at the mRNA level in breast cancers but have not been systematically validated at the protein level. Based on an extensive literature search, we identified ribosome binding protein 1 (RRBP1) as a candidate that is upregulated at the mRNA level in five different studies but its protein expression had not been investigated. Immunohistochemical labeling of breast cancer tissue microarrays was carried out to determine the expression of RRBP1 in a large panel of breast cancers. We found that RRBP1 was overexpressed in 84% (177/219) of breast carcinoma cases tested. The subcellular localization of RRBP1 was mainly observed to be in the cytoplasm with intense staining in the perinuclear region. Our findings suggest that RRBP1 is an interesting molecule that can be further studied for its potential to serve as a breast cancer biomarker. This study also demonstrates how the integration of biological data from available resources in conjunction with systematic evaluation approaches can be successfully applied to clinical proteomics.

## Background

Breast cancer is the most prevalent cancer in women and is responsible for ~450,000 deaths worldwide each year. It accounted for 23% of new cancer cases and 14% of total cancer deaths in 2008 [1]. The molecular pathology of breast cancer has been studied extensively over the last two decades and a large number of alterations at the molecular level have been reported by several groups. Gene expression profiling studies are a powerful means of investigating changes in the transcriptome of cancerous cells. They provide a broad view of the numerous candidate genes that are differentially expressed in normal and cancerous lesions [2]. Many of these genes show significant changes in corresponding protein expression. Large scale gene expression profiling studies have been carried out on various subtypes of breast carcinomas by different groups [3-5]. Although these studies have reported many common genes that are significantly upregulated at the mRNA level in breast tumors, such findings are not of much importance if the overexpression is not reflected at the protein level. Moreover, high-throughput techniques like microarrays are associated with limitations such as experimental and biological noise that necessitate further validation of the results obtained using other relatively accurate methods. The current rush of RNA-Seq analyses will also require the same kind of validation that mRNA transcripts are actually translated into proteins.

Our approach in this study was to integrate data from public repositories of gene expression information and online resources of protein information coupled to literature mining to generate a list of molecules that have been shown to be overexpressed at the transcript level but not at the protein level. We selected RRBP1 as one such candidate to further validate its protein expression across a panel of breast carcinomas using immunohistochemistry.

RRBP1(Q9P2E9, ENSP00000367044.1) is an endoplasmic reticulum membrane protein [6-8] that is essential for ribosome binding and for the translocation of nascent proteins across the membrane of the rough endoplasmic reticulum [9]. Although RRBP1 is known to be localized primarily to the endoplasmic reticulum, it has also been detected in the nucleus and the cytoplasm [10]. It is a 1410 amino acid protein composed of a hydrophobic NH_2_-terminus that includes a transmembrane domain, a highly conserved tandem repeat (ribosome-binding domain), and an acidic coiled-coil COOH-terminal domain [11]. It has been shown to play an important role in pro-collagen biosynthesis in secretory tissues and in the terminal differentiation of secretory tissues [6,12]. It has also been shown to interact with KIF5B [13], a motor protein highly expressed in several cancer cell lines [14]. There are limited reports on expression of this protein in cancers. In a study by Krasnov *et al.*, it has been shown to be overexpressed in colorectal cancers [15].

Immunohistochemistry is a robust method to validate the differential expression of proteins at tissue level. Tissue microarray arrays (TMAs) allow the testing of a single biomarker in a high-throughput fashion to test large number of normal and cancerous tissues simultaneously. Using this approach, we could identify the overexpression of RRBP1 in 84% of the breast tumors assayed. RRBP1, as a consequence of its overexpression, could possibly leak into blood plasma and show up at detectable levels in breast carcinomas. We foresee that RRBP1 could be a promising biomarker if validated in a larger cohort, especially in the plasma or serum.

## Results and discussion

### Selection of RRBP1 as a candidate marker for evaluation

To identify novel candidate biomarkers for breast cancers, we collected information about genes that have been reported to have significantly altered expression levels in breast tumors. We used the online gene expression database Oncomine [16,17] to generate a list of genes which were transcriptionally upregulated in breast cancer. We next checked the published literature to determine if they have been studied for their expression at the protein level. The Human Protein Reference Database (HPRD) [18-20] was next used to obtain additional information on the molecules that were short-listed. Out of the list of genes that were transcriptionally upregulated in breast cancers but were not yet reported to be overexpressed at the protein level, the top ten candidates of which are shown in Table 1, we chose RRBP1 for further evaluation. We found five published studies that reported significant upregulation of *RRBP1* at the transcript level. A summary of results of the query we made in Oncomine is shown in Table 2.

**Table 1:** The top ten shortlisted molecules with reports of overexpression at transcript level but with no protein level studies in breast cancers

	**Gene symbol**	**Description**	**Number of studies in Oncomine reporting significant overexpression in breast cancers**
1	*RRBP1*	Ribosome binding protein 1 homolog 180 kDa	5
2	*XPNPEP3*	X-prolyl aminopeptidase (aminopeptidase P) 3, putative	5
3	*RCOR3*	REST corepressor 3	5
4	*CDS2*	CDP-diacylglycerol synthase (phosphatidate cytidylyltransferase) 2	4
5	*LSR*	Lipolysis stimulated lipoprotein receptor	4
6	*PDE4DIP*	Phosphodiesterase 4D interacting protein	4
7	*PGPEP1*	Pyroglutamyl-peptidase I	3
8	*C9ORF46*	Transmembrane protein C9orf46	2
9	*KIFAP3*	Kinesin-associated protein 3	2
10	*ZYG11A*	Zyg-11 homolog A	2

**Table 2:** Studies from ONCOMINE database showing significant upregulation of *RRBP1* in breast cancer vs normal analyses

	**Study**	**Sample size**	**Analysis type**	**Fold change**	**p-value**
1.	Radvanyi *et al.* (2005) [21]	63	Ductal Breast Carcinoma in Situ vs. Normal	3.3	p = 0.017
			Invasive Mixed Breast Carcinoma vs. Normal	2.4	p = 0.013
			Invasive Ductal Breast Carcinoma vs. Normal	2.2	p = 0.021
2.	Finak *et al.* (2009) [22]	59	Invasive Breast Carcinoma Stroma vs. Normal	1.8	p = 4.75E-5
3.	Perou *et al.* (2000) [23]	65	Lobular Breast Carcinoma vs. Normal	1.7	p = 0.046
4.	Zhao *et al.* (2004) [24]	64	Invasive Ductal Breast Carcinoma vs. Normal	1.7	p = 0.007
			Lobular Breast Carcinoma vs. Normal	1.4	p = 0.022
5.	Turashvili *et al.* (2007) [25]	30	Invasive Ductal Breast Carcinoma vs. Normal	1.3	p = 0.007

RRBP1 was chosen as a candidate because of the availability of an antibody in the Human Protein Atlas (HPA) [26,27] that worked in immunohistochemistry applications and also because of it known role in tumorigenesis [15].

We next performed immunohistochemistry on tissue microarrays to test the expression of RRBP1 protein across normal breast epithelium, benign hyperplasias, in situ carcinomas, invasive tumors and also metastatic breast cancers.

### RRBP1 overexpression in breast tumors

**Figure 1:**
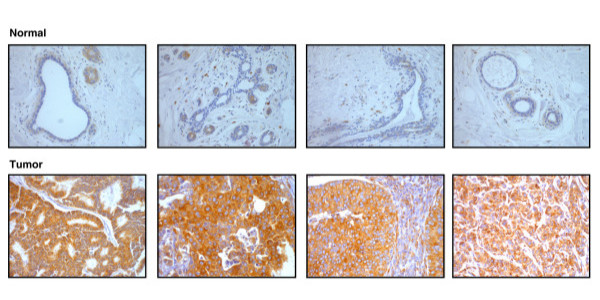
**Expression pattern of RRBP1 protein in normal and breast cancer tissues.** The images shown here are of the tissue sections from TMAs stained with anti-RRBP1. The panel of four sections on the top represents normal breast tissue sections showing weak expression of RRBP1 protein. The lower panel represents four tumor sections that show significant overexpression of the protein. All the images were taken at 200X magnification.

Tissue microarrays were immunohistochemically labeled using a polyclonal antibody directed against a unique region of RRBP1. The immunogen used was a recombinant peptide of 107 amino acids that constitutes a part of a coiled coil motif on the protein [28]. Figure 1 shows the images of some anti-RRBP1 stained normal and breast cancer tissue sections. The differential expression of the protein among the normal breast tissue sections (upper panel) and the tumor tissue sections (lower panel) can be clearly visualized. We used a progression TMA to assess the expression of this protein in diverse types of benign breast tumors and malignant breast carcinomas. We observed that the expression of RRBP1 protein showed a definite increase in malignant tumors when compared to normal, benign and hyperplastic conditions. However, we did not observe any gradation in the expression of RRBP1 in benign or hyperplastic conditions. Table 3 shows the pattern of expression of RRBP1 across the cases tested. As shown in the table, 84% of the tumors showed overexpression of RRBP1. The expression of RRBP1 in the 16 controls tested showed a weak to moderate cytoplasmic staining. Out of the 219 cases tested, 177 cases showed positive staining in carcinoma cells alone, 25 cases showed positive staining in tumor as well as the stromal components and 16 cases showed overexpression only in the stroma but not in the tumor cells.

**Table 3:** Summary of RRBP1 expression pattern in breast cancer tissues

	**Parameters**	**Number of cases**
1.	Number of cases tested	259
2.	Number of positive cases	219
3.	Number of cases with positive staining of carcinoma cells alone	177
4.	Number of cases with positive staining of stroma alone	16
5.	Number of cases with positive staining of both carcinoma and stromal component.	25

### Public availability and accessibility of IHC data

**Figure 2:**
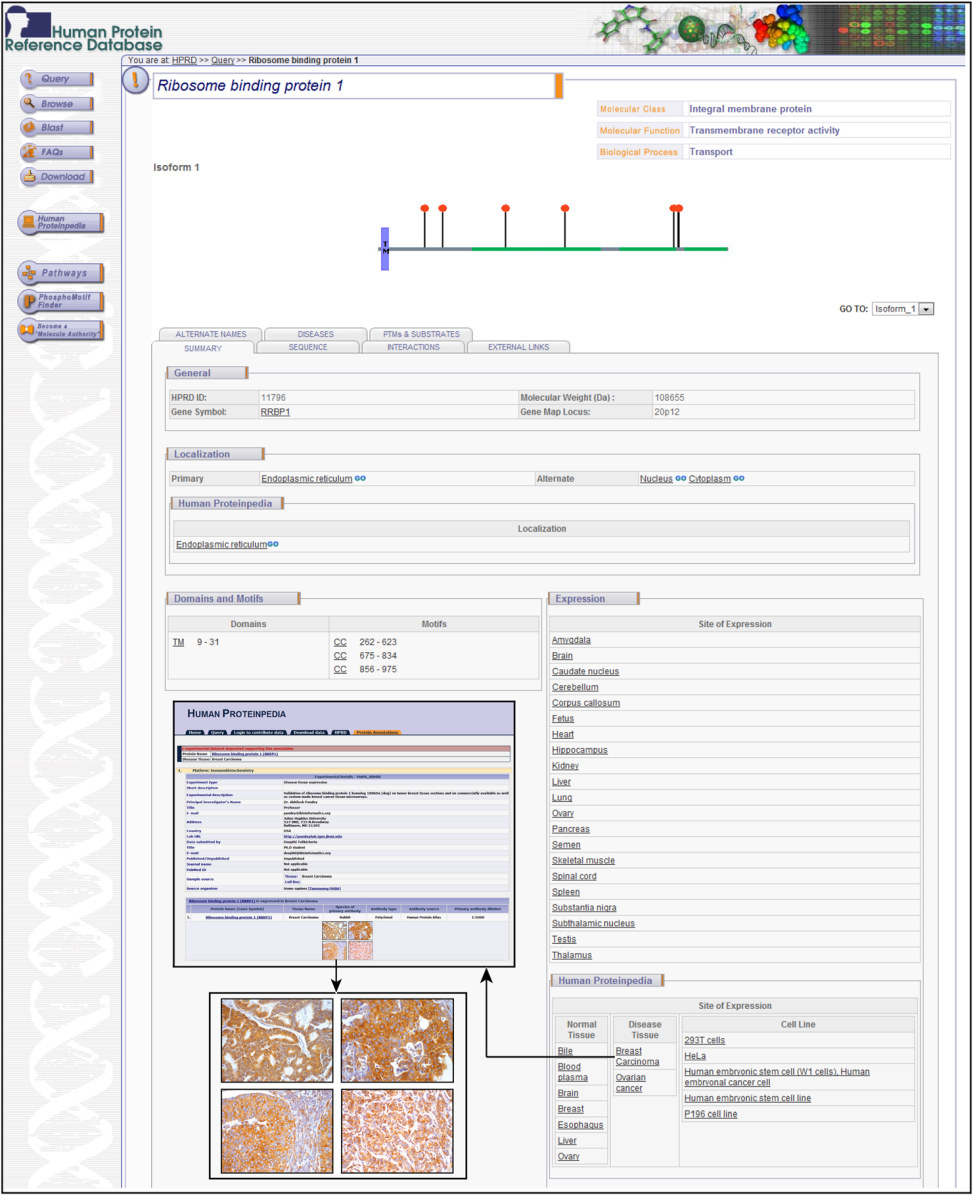
**A snapshot of RRBP1 annotation in human Proteinpedia.** Shown here is a screenshot of the HPRD molecule page for RRBP1 protein depicting the gene information, architecture, post-translational modifications, cellular localization and tissue expression of the molecule. The figure also shows the immunohistochemical data from this study as displayed in HUPA in the inset.

To make our observations publicly available and accessible to other researchers, we have submitted our data on the immunohistochemical analysis of RRBP1 to Human Proteinpedia (HUPA) [29,30]. The expression of RRBP1 in normal breast epithelium [31] and in breast cancer [32] can be visualized in separate pages. Figure 2 shows a screenshot of HPRD molecule page for RRBP1 and also of HUPA resource which is linked to HPRD, displaying RRBP1 expression in breast cancer.

## Conclusions

The results we obtained from our experiment show that RRBP1 was significantly upregulated in breast cancers. The unique work plan we followed to close in on RRBP1 protein using online gene expression data repositories, databases that provide protein information and published literature has proven beneficial in finding candidate biomarkers in a fast and effective manner. This study highlights the power of a bioinformatics approach coupled to experimental validation. We found RRBP1 as a novel candidate marker that is significantly overexpressed in invasive breast carcinomas. However, further analyses across larger sample sets and insights into the functional aspects of the molecule are necessary to confirm its significance as a breast cancer biomarker.

## Methods

### Tissue microarrays

We used two types of TMAs including one breast cancer progression TMA (n = 36) that was purchased from Creative Biolabs (TMA-029) and custom TMAs (n = 44) that were prepared by JCR. The custom TMA construction and the use of cases was approved by the Institutional Review Board (IRB) at the Universidad de La Frontera, Temuco, Chile.

### Immunohistochemical staining

Immunohistochemical staining was performed on breast cancer tissue microarrays for RRBP1. The rabbit polyclonal antibody against RRBP1 was purchased from the Human Protein Atlas (catalog # HPA009026). Anti-RRBP1 antibody was used at 1:5000 dilution. An Envision kit (DAKO) was used following the manufacturer’s instructions. Briefly, tissue microarrays were deparaffinized and antigen retrieval was carried out by incubating for 20 minutes in antigen retrieval buffer. Endogenous peroxidases were quenched using the blocking solution followed by three washes with the wash buffer. The sections were incubated overnight at 4°C in a humidified chamber with the primary antibody. After washing, the slides were incubated with appropriate horseradish peroxidase conjugated secondary antibody for 30 minutes at room temperature. Immunoperoxidase staining was developed for 5 minutes using DAB chromogen, and counterstained with hematoxylin (Nice Chemicals, India). We used the diluent instead of primary antibody as the negative control. The images were taken by using LEITZ DMRB model (Leica Microsystems, Wetzlar, Germany) microscope at a magnification of 200X. Immunohistochemical labeling was assessed by an experienced expert pathologist (JM), and intensity of staining was scored as negative (0), mild (1+), moderate (2+), or strong (3+). The distribution of staining of cancer cells was scored as 0 (less than 5% of cells staining), 1+ (5%–30% of cells staining), 2+ (31%–60% of cells staining) or 3+ (greater than 60% of cells staining). Comparisons were made between the intensity of the staining of carcinoma cells and that of normal breast tissue.

## Abbreviations

RRBP1, Ribosome binding protein 1; TMA, Tissue microarray; KIF5B, Kinesin family member 5B.

## Competing interests

The author(s) declare that they have no competing interests.

## Authors’ contributions

AP designed the study. DT, AM and MKK did the bioinformatics analysis for selection of candidate molecule and also performed the immunohistochemical staining. JCR provided the breast cancer TMAs. JM scored the stained tissue sections. SM and YLR participated in design and coordination of the study and helped to draft the manuscript. All authors reviewed and edited the manuscript. All authors read and approved the final manuscript.
